# Baicalin Protects against Thrombin-Induced Cell Injury in Human Umbilical Vein Endothelial Cells

**DOI:** 10.1155/2019/2187306

**Published:** 2019-08-06

**Authors:** Anna Zhang, Yunfeng Hou, Chao Sun, Ying Pu, Yu Sun, Yuan Zhang, Yang Shen, Qingbo Zhou

**Affiliations:** ^1^Department of Geriatrics, Southern Hospital of the Second Hospital of Shandong University, Jinan 250033, China; ^2^Intensive Care Unit, Qianfoshan Hospital, Shandong University, Jinan 250014, China; ^3^Central Laboratory, The Second Hospital of Shandong University, Jinan 250033, China; ^4^Electromyography Lab, Jinan Central Hospital, Jinan 250033, China; ^5^Department of Pharmacology, School of Basic Medical Science, Shandong University, Jinan 250012, China; ^6^Center of Evidence-Based Medicine, The Second Hospital of Shandong University, Jinan 250033, China; ^7^Department of Neurology, The Second Hospital of Shandong University, Jinan 250033, China

## Abstract

Thrombin plays a pivotal role in the pathogenesis of atherosclerosis. Baicalin, an active flavonoid compound, was shown to attenuate the development of atherosclerosis, but the mechanism remains elusive. In the present study, the role and mechanism of baicalin in thrombin-induced cell injury was investigated in human umbilical vein endothelial cells (HUVECs). Our results showed that baicalin significantly reduced thrombin-induced apoptosis of HUVECs. Additional experiments showed that baicalin inhibited thrombin-induced NF-*κ*B activation and PAR-1 expression. In addition, baicalin decreased thrombin-induced PAR-1 expression by inhibiting ERK pathway. These results indicated that baicalin has protective effects on thrombin-induced cell injury in HUVECs possibly through inhibition of PAR-1 expression and its downstream NF-*κ*B activation, which was mediated by ERK1/2 activation.

## 1. Introduction

Atherosclerosis is the major cause of cardiovascular disease (CVD). Although great progress has been made, the incidence of CVD is increasing and the patients with atherosclerosis are becoming younger [1]. Since 1990, CVD has become the leading cause of death in China [2].

The development of atherosclerosis involves in a variety of biological processes, including vascular endothelial cell injury, dyslipidemia, macrophage and T cell infiltration, smooth muscle cell hyperplasia, and thrombosis. Recently, accumulating evidence suggested that thrombin may play a pivotal role in the pathogenesis of atherosclerosis via damage of vascular endothelial cells [1].

Thrombin is produced by the enzymatic cleavage of two sites on prothrombin. Thrombin interacts with its specific cell membrane receptors (protease activated receptors: PAR-1, PAR-3, and PAR-4) which are abundantly expressed in all arterial vessel wall constituents, to participate in a number of biological processes, such as inflammation, leukocyte recruitment, oxidative stress, migration, and proliferation of vascular smooth muscle cells, cell apoptosis, and angiogenesis [3–5]. Transcription factor NF-*κ*B plays an essential role in inflammation. NF-*κ*B was shown to be activated upon thrombin stimulation and contributed to cell injury [6, 7].

Baicalin (7-glucuronic acid, 5, 6-dihydroxyflavone) is an active flavonoid compound, which is isolated from the medicinal plant Scutellaria baicalensis Georgi. It possesses many biological properties including antioxidant, antiplatelet, antithrombotic, and anticarcinogenic activities [8, 9]. Therefore, baicalin has been widely used for treatment of inflammatory diseases, such as asthma, nephritis, hepatitis, bronchitis, atopic dermatitis, and acute ischemic cerebrovascular diseases [10–12]. Our previous studies showed that baicalin had protective effects on SH-SY5Y cells against thrombin-induced cell injury through inhibiting the expression of PAR-1, MMP-9, and NMDA-R1 [13] and attenuated focal cerebral ischemia reperfusion injury in an acute cerebral ischemia rat model [14]. Additionally, baicalin was also suggested to ameliorate atherosclerosis possibly through lipid regulation, immune regulation, or activation of Wnt signaling pathway [15–17]. However, it is unclear whether baicalin has a protective effect on thrombin-induced cell injury in human umbilical vein endothelial cells (HUVECs).

In this study, the effects of baicalin on thrombin-induced cell injury in HUVECs were examined, and the underlying mechanisms were investigated.

## 2. Materials and Methods

### 2.1. Reagents

Thrombin (T6884) was purchased from Sigma-Aldrich (St. Louis, MO, USA). Baicalin was purchased from Haotian Pharmaceutical Company (Chinese Drug Approval No. H20073931, 95% purity, Weifang, China) and dissolved in dimethyl sulfoxide. 3-(4,5-dimethylthiazol-2-yl)-2,5-diphenyltetrazolium bromide (MTT) was purchased from Amresco (OH, USA). Annexin V-FITC/PI Apoptosis Detection Kit was obtained from BD Pharmingen. Caspase-3 assay kit was purchased from Sigma-Aldrich (St. Louis, MO, USA). Trizol RNA-RNAiso Plus, Prime Script RT reagent kit with gDNA Eraser, and SYBR premix Ex Tap were purchased from TakaRa (Dalian, China). Antibodies against PAR-1, caspase-3, P65, p-P65, ERK1/2, p-ERK1/2, and GAPDH were obtained from Abcam (Cambridge, UK). Goat anti-rabbit IgG antibody was obtained from GenScript (Nanjing, China).

### 2.2. Cell Lines and Cell Culture

HUVECs were obtained from American Type Culture Collection (ATCC, Manassas, VA, USA) and maintained in RPMI-1640 (Corning) supplemented with 10% fetal bovine serum (FBS, Gibco), penicillin G (100 U/mL), and streptomycin (100 *μ*g/mL) in a 5% CO_2_ incubator at 37°C. Cells of the 4-8 generation were used in all experiments. In experiments for thrombin stimulation, HUVECs were pretreated with baicalin at different concentrations (0 *μ*M, 50 *μ*M, 100 *μ*M, and 150 *μ*M) for 3 hours, followed by treatment with thrombin (5 U/mL) for additional 6 hours.

### 2.3. Cell Proliferation Assay

HUVECs were seeded in 96-well plates at a density of 1 × 10^4^ cells per well and treated with baicalin and thrombin. The MTT solution was added to each well at a concentration of 5 mg/mL and the cells were cultured for additional 4 hours at 37°C. Supernatants were removed and replaced with 150 *μ*L dimethyl sulfoxide (DMSO) into each well. Ten minutes later, the absorbance of each well was measured at 490 nm using a microplate reader.

### 2.4. Cell Apoptosis Assay

HUVECs were seeded in 6-well plates at a density of 2 × 10^5^ cells per well and treated with baicalin and thrombin. Next, cells and supernatants were collected, washed with phosphate buffered solution (PBS), and resuspended in 100 *μ*L1 × binding buffer. Then 5 *μ*L of FITC-labelled Annexin V and PI were added and incubated at room temperature in the dark. After 15 minutes, 400 *μ*L1 × binding buffer was added. Cell apoptosis was analyzed using flow cytometry.

### 2.5. Measurement of Caspase-3 Activity

HUVECs were treated with baicalin and thrombin. According to the manufacturer's instructions, the activity of caspase-3 like protease in pyrolysis solution was evaluated by using colorimetric caspase-3 assay kit. In brief, the reaction mixture (total volume, 100 *μ*L) including 30 *μ*L of cell lysate and 10 *μ*L of caspase-3 substrate acetyl–Asp–Glu–Val–Asp–p-nitroanilide (final concentration at 200*μ*M) was used and determined on 96-well plate. The mixture was incubated for 90 minutes at 37°C and the absorbance was measured at 405 nm. The caspase-3 activity was expressed by value of OD405 relative to control.

### 2.6. RNA Extraction and Quantitative Real-Time Polymerase Chain Reaction

Total RNA was extracted purified using Trizol RNA-RNAiso Plus. Reverse transcription was performed to generate complementary DNA (cDNA) using Prime Script RT reagent kit with gDNA Eraser according to manufacturer's protocol. The mRNA expression of PAR-1 was measured by quantitative real-time polymerase chain reaction using SYBR premix Ex Tap TM (TLiRNSEH PLUS). Predesigned primers were as follows: *β*-actin forward, 5′-TGACGTGGACATCCGCAAAG-3′; *β*-actin reverse, 5′-CTGGAAGGTGGACAGCGAGG-3′; PAR-1 forward, 5′-CACAAACGTCCTCCTGATTG-3′; PAR-1 reverse, 5′-ATGCTGCTGACACAGACACA-3′; *β*-actin was used as internal control. The relative mRNA expression of these genes was calculated using the 2^(−ΔΔCt)^ method.

### 2.7. Western Blotting

Cells were lysed with lysis buffer containing protease inhibitors and NP-40, and the protein concentration was quantified using Bicinchoninic acid (BCA) assay. Next, the protein samples (40 *μ*g per lane) were subjected to SDS-PAGE and transferred to a polyvinylidene fluoride membrane. The membrane was blocked in 5％ nonfat milk at room temperature for an hour and then incubated with antibodies at 4°C overnight. The membrane was washed 3 times with PBS containing 0.5％ Tween-20 and incubated with corresponding horseradish peroxidase-conjugated secondary antibody for 2 hours. The blots were visualized using enhanced chemiluminescence (Perkin Elmer, Boston, MA, USA).

### 2.8. Statistical Analysis

All data are shown as the mean ± standard deviation (SD). Statistical differences between the groups were determined by repeated measurement analysis of variance tests. All statistical analyses were conducted using GraphPad Prism 5.0 (GraphPad Software Inc., San Diego, CA, USA). A value of* P*< 0.05 was considered statistically significant.

## 3. Results

### 3.1. Effect of Baicalin on Thrombin-Induced Inhibition of Cell Survival

**Figure 1:**
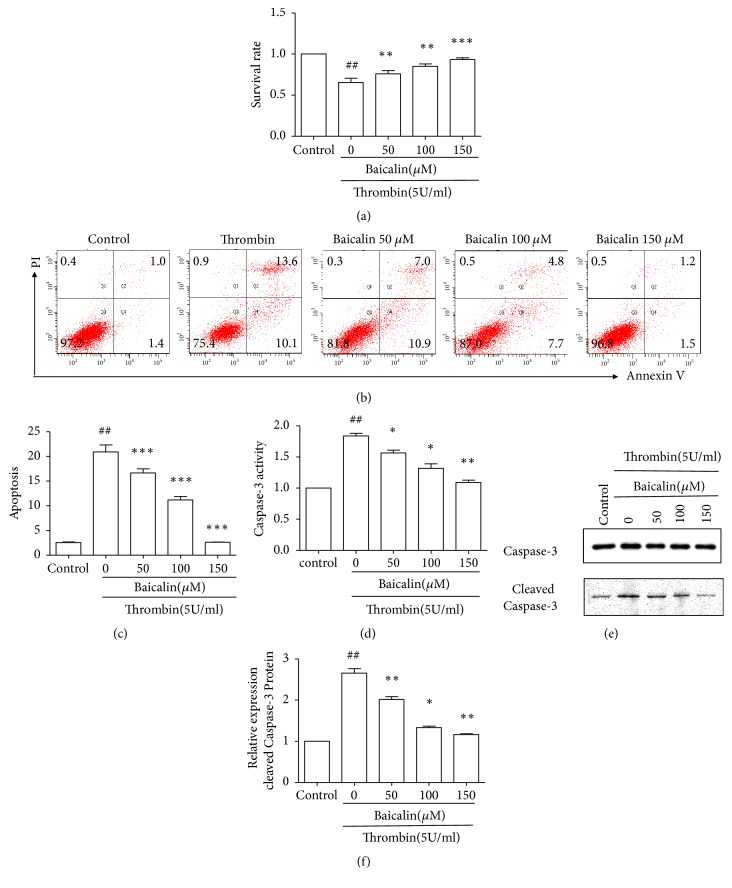
*Effects of baicalin on cell survival and apoptosis after thrombin treatment*. HUVECs were pretreated with baicalin (0, 50, 100, and 150*μ*M) for 3 hours before exposure to thrombin (5 U/ml) for 6 hours. (a) Cell viability was determined by MTT assay. (b) Apoptosis was determined by flow cytometry. (c) Rate of apoptotic cells quantified of three independent experiments by flow cytometry was calculated. (d) Caspase-3 activity was determined by a caspase-3 assay kit. Caspase-3 activity relative to control that was set as 1. (e) Representative experiments of western blot for the cleaved caspase-3 protein expression. (f) Quantitative analysis of the ratio of cleaved caspase-3 to caspase-3. Values are means ± SD (n=3). ## P<0.01 vs. control group; *∗*P<0.05; *∗∗*P<0.01; *∗∗∗*P<0.001 vs. thrombin group.

First, the effect of baicalin on thrombin-induced cell damage was evaluated in HUVECs. Cell viability was analyzed by MTT assay. HUVECs were pretreated with baicalin at different concentrations (0 *μ*M, 50 *μ*M, 100 *μ*M, and 150 *μ*M) for 3 hours, followed by treatment with thrombin (5 U/ml) for additional 6 hours. As shown in Figure 1(a), compared with control cells, cell viability was significantly decreased in HUVECs treated with thrombin. The inhibitory effect of thrombin on cell viability was partially abolished by pretreatment with baicalin in a dose-dependent manner.

### 3.2. Inhibitory Effect of Baicalin on Thrombin-Induced Cell Apoptosis

As MTT assay could not make a distinction between cells undergoing necrosis or apoptosis, flow cytometry was performed to determine cell apoptosis. As shown in Figures 1(b) and 1(c), compared with control cells, the percentage of apoptotic cells increased in HUVECs treated with thrombin. Pretreatment with baicalin decreased thrombin-induced cell apoptosis in a dose-dependent manner.

To verify the above results, the activity of caspase-3 was determined by using a caspase-3 assay kit, and the levels of caspase-3 and cleaved caspase-3 protein were examined by western blotting. As shown in Figure 1(d), compared with control cells, thrombin induced caspase-3 activation, which was inhibited by baicalin in a dose-dependent manner. As shown in Figures 1(e) and 1(f), western blot analysis revealed a higher level of cleaved caspase-3 in thrombin treated cells than control cells. Accordingly, baicalin decreased the level of cleaved caspase-3.

### 3.3. Inhibitory Effect of Baicalin on Thrombin-Induced NF-*κ*B Activation

**Figure 2:**
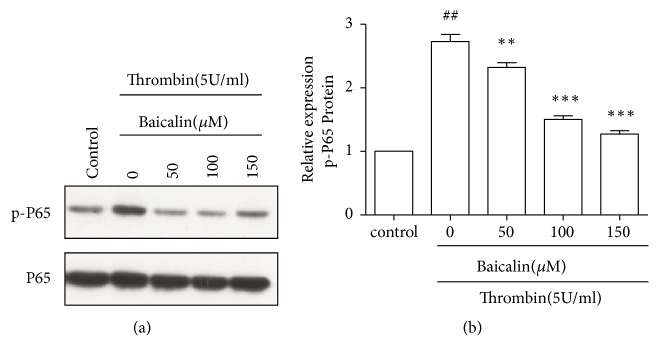
*Baicalin reduced thrombin-induced NF-κB activation*. Cells were treated as indicated and then were lysed for western blotting analysis. (a) Representative results of western blotting experiments to detect p-P65, P65 was used as a loading control. (b) Quantitative analysis of the ratio of p-P65 to P65. Values are means ± SD (n=3). ## P<0.01, vs. control group; *∗∗* P<0.01; *∗∗∗* P<0.001 vs. thrombin group.

NF-*κ*B was activated upon thrombin stimulation and contributed to cell injury in previous study. In the present study, we evaluated NF-*κ*B activation by detection of P65 phosphorylation. The expression of NF-*κ*B (P65) and p-P65 was determined by western blotting. As shown in Figures 2(a) and 2(b), the expression of p-P65 was significantly increased in HUVECs treated with thrombin compared with control cells, which was attenuated by baicalin in a dose-dependent manner.

### 3.4. Inhibitory Effect of Baicalin on Thrombin-Induced PAR-1 Expression

**Figure 3:**
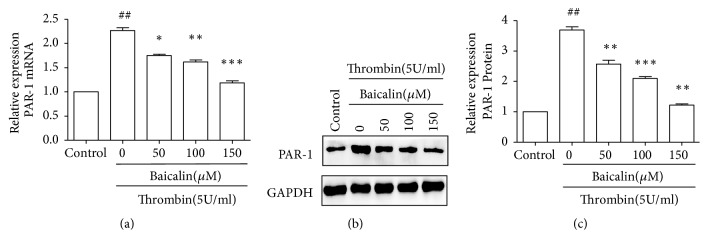
*Baicalin suppressed thrombin-induced the PAR-1 expression*. Cells were treated as indicated, then total RNA was extracted for real-time RT-PCR analysis, and cells were lysed for western blotting analysis. (a) The quantitation represents the average relative ratio of PAR-1 mRNA to *β*-actin. (b) Representative experiments of western blot for the PAR-1 protein expression. (c) The quantitation represents the average relative ratio of PAR-1 protein to GAPDH. Values are means ± SD (n=3). ## P<0.01 vs. control group; *∗*P<0.05, *∗∗*P<0.01, and *∗∗∗*P<0.001 vs. thrombin group.

PAR-1 was shown to participate in the processes of cell apoptosis. Thus, we examined the effect of baicalin on thrombin induced PAR-1 expression. The expression of PAR-1 in mRNA and protein levels was determined by RT-PCR and western blotting, respectively. As shown in Figure 3, the expression of PAR-1 was significantly increased in mRNA and protein levels after thrombin treatment, which was partly inhibited by baicalin in a dose-dependent manner.

### 3.5. Baicalin Decreased Thrombin-Induced PAR-1 Expression through Inhibition of ERK1/2 Phosphorylation

**Figure 4:**
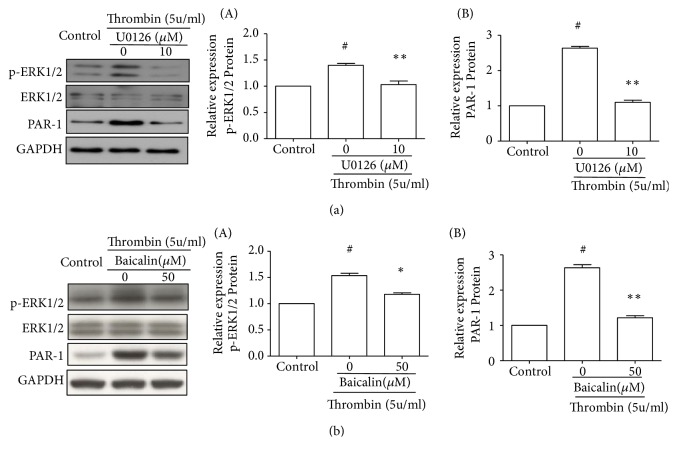
*Baicalin decreased thrombin-induced PAR-1 expression through inhibition of ERK1/2 phosphorylation*. (a) HUVECs were pretreated with U0126 (10*μ*M) for 3 hours before exposure to thrombin (5 U/ml). (b) HUVECs were pretreated with baicalin (50*μ*M) for 3 hours before exposure to thrombin (5 U/ml). Representative experiments of western blot for the p-ERK1/2, ERK1/2, and PAR-1 protein expression. (A) Quantitative analysis of the ratio of p-ERK1/2 to ERK1/2, ERK1/2 was used as a loading control. (B) Quantitative analysis of the ratio of PAR-1 to GAPDH. Values are means ± SD (n=4). #P<0.05 vs. control group; *∗*P<0.05; *∗∗*P<0.01 vs. thrombin group.

To investigate the mechanism of baicalin inhibiting PAR-1 expression, we detected the phosphorylation of ERK1/2. HUVECs were pretreated with U0126 (10 *μ*M) for 3 hours before exposure to thrombin (5 U/ml). As shown in Figure 4(a), thrombin exposure in HUVEC induced a rapid ERK1/2 phosphorylation, which was partly abrogated by pretreatment of U0126 (10 *μ*M), a selective inhibitor of MAP kinase. Similarly, pretreatment with baicalin (50 *μ*M) also partly inhibited thrombin-induced ERK1/2 phosphorylation as shown in Figure 4(b). Both U0126 and baicalin decreased thrombin-induced PAR-1 expression.

## 4. Discussion

In this study, we demonstrated that baicalin significantly inhibited thrombin-induced upregulation of cell apoptosis in HUVECs. The major mechanisms included inhibition of PAR-1 expression and inhibition of MAP kinase, NF-*κ*B, and caspase-3 activation.

Endothelial cells apoptosis occurs throughout the early stages of atherosclerosis and plays important roles in plaque regression and plaque instability [18, 19]. Apoptosis, or programed cell death, is a serious form of endothelial cells injury and fundamental to physiologic processes [20]. The signaling pathways leading to apoptosis can generally be divided into the extrinsic (surface receptor-mediated) and intrinsic (mitochondrial dependent) pathways [21]. Apoptotic pathways can be modulated by various inflammatory molecules and elucidation of these mechanisms may lead to novel therapeutic interventions that inhibit key steps in the apoptosis pathways. Apoptosis is tightly regulated and primarily carried out by aspartate-specific cysteine proteases termed “caspases”. As is known to all, caspase-3 cleavage is the main hallmark of apoptosis [22]. In this study, compared with cells in the control group, the percentage of apoptotic cells increased in HUVECs treated with thrombin, and pretreatment with baicalin decreased thrombin-induced cell apoptosis in a dose-dependent manner. We found that baicalin inhibited the thrombin-induced caspase-3 activation in a dose-dependent manner.

The expression of thrombin was elevated in patients with ischemic cerebrovascular disease (CVD) in the previous reports [14, 23]. Protease activated receptors, which are abundantly expressed in all arterial vessel wall, are a family of G-protein coupled receptors comprising four members (i.e., PAR-1, PAR-2, PAR-3, and PAR-4) [24]. However, thrombin interacts with PAR-1 to participate a number of biological processes, such as inhibition of cell proliferation, induction of cell apoptosis, activation of inflammation and upregulation of adhesion molecules and matrix metalloproteinase (MMP) [25, 26]. Previous studies reported that the expression of PAR-1 was upregulated after thrombin stimulation in rodent models [27]. PAR-1 activation by thrombin has been found to activate or inhibit apoptosis in neuronal cells, astrocytes, endothelial cells, epithelial cells, fibroblasts, and tumor cells in a dose-dependent fashion [28–30]. In addition, it has been found that PAR-1 activation can increase epithelial cell apoptosis and intestinal permeability in a caspase-3 dependent manner [30]. Our results showed that PAR-1 was dramatically upregulated after thrombin stimulation in HUVECs, while baicalin inhibited the expression of PAR-1 in a dose-dependent manner.

Nuclear factor (NF)-*κ*B signaling was associated with the transcriptional regulation of various genes involved in inflammatory responses, cell growth, survival, and apoptosis [31, 32]. In unstimulated cells, NF-*κ*B binds to inhibitory molecule I*κ*B which retains NF-*κ*B in an unstimulated state and prevents its migration to nucleus. Upon stimulated, I*κ*B is degraded, releasing NF-*κ*B that translocates to the nucleus and promotes target genes transcription [33]. It can be found that NF-*κ*B can be activated upon thrombin stimulation and caused cell injury [7, 34]. The importance of NF-*κ*B signaling in regulating the apoptotic program has been demonstrated in various cells [32]. It can be demonstrated that NF-*κ*B signaling is crucial in preventing heat stress-induced apoptosis of HUVECs [35]. It was also found that activated HUVECs are involved in atherosclerosis through the activation of NF-*κ*B and release inflammatory factors [36]. It has been suggested that PAR-1 causes apoptosis of cells to be regulated by activation of NF-*κ*B [37, 38]. Baicalin attenuated thrombin-induced upregulation of NF-*κ*B in SH-SY5Y cells [13]. Consistent with previous studies, we demonstrated that baicalin inhibited thrombin-induced activation of NF-*κ*B in a dose-dependent manner in HUVECs.

Baicalin is a kind of Chinese traditional medicine, which has multiple potential biological functions anti-inflammatory and antiapoptosis [39, 40]. However, the biotransformation of baicalin is poorly understood. It is due in part to difficulties that have been encountered in obtaining enough amounts to identify the structure of the metabolites and study the bioactivities of them [41]. To explore the mechanism of baicalin in inducing thrombin-induced cell apoptosis in HUVECs, we studied the effect of baicalin on the PAR-1 expression and NF-*κ*B activation. In the present study, we found that thrombin upregulated PAR-1 expression and then activated NF-*κ*B pathway, which caused cell apoptosis in HUVECs. Baicalin protected against thrombin-induced cell injury by inhibiting the expression of PAR-1 and NF-*κ*B activation.

Increasing evidence suggests that activation of the ERK signaling pathway is also involved in inflammation and apoptosis [42, 43]. Thrombin-mediated PAR-1 gene expression in endothelial cells requires the heterotrimeric Gi-activated Ras/MAPK signaling pathway [44]. Besides, ERK signaling pathway plays a key role in baicalin protection against oxygen-glucose deprivation induced rat brain microvascular endothelial cells injury and acetaminophen-induced liver injury [45, 46]. To get more insight into the mechanism of baicalin on thrombin-induced cell injury, we examined the effect of baicalin on ERK1/2 activation. Our results showed that thrombin-induced PAR-1 expression was accompanied with concomitant activation of ERK1/2. U0126, a specific inhibitor of ERK1/2, prevented thrombin-induced ERK1/2 activation and partly inhibited thrombin-induced PAR-1 expression. These results suggested that thrombin-induced PAR-1 expression is at least partly mediated by ERK1/2 activation. Further experiments showed that baicalin had a similar effect as U0126. Pretreatment with baicalin partly inhibited thrombin-induced ERK1/2 activation and PAR-1 expression. We proposed that baicalin can inhibit PAR-1 expression by inhibiting ERK pathway. In future, further studies are required to investigate the mechanism by which baicalin prevents ERK1/2 activation.

## 5. Conclusion

In conclusion, our study showed that baicalin has protective effects on thrombin-induced cell injury in HUVECs possibly through inhibition of PAR-1 expression and its downstream NF-*κ*B activation, raising the possibility of use of baicalin as a drug for the prevention and treatment of atherosclerosis. Baicalin could inhibit thrombin-induced PAR-1 expression, which was mediated by ERK1/2 activation.

## Data Availability

The data used to support the findings of this study are included within the article.
